# ‘We only got Coca-Cola’: Disability and the paradox of (dis)empowerment in Southeast Nigeria

**DOI:** 10.4102/ajod.v8i0.444

**Published:** 2019-04-25

**Authors:** Okechukwu V. Nwokorie, Patrick J. Devlieger

**Affiliations:** 1 Interculturalism, Migration and Minorities Research Centre (IMMRC), Faculty of Social Sciences, University of Leuven, Leuven, Belgium

**Keywords:** mobility disability, empowerment paradox, survival strategy, empowerment discourse, culture, empowerment, Igbo culture, local and global, power elites

## Abstract

**Background:**

Empowerment is the generic name for support services for persons with disability in Nigeria. In it, the elites of the society play leading roles. Special events such as anniversaries, Christmas seasons, wealthy people’s birthdays, investiture of new titles and campaigns before general elections often provide occasions for empowerment programmes.

**Objectives:**

This article explores discourses of empowerment of persons with disability in Southeast Nigeria. We concentrate on the relation between local elites and the disability community and how it impacts our understanding of empowerment. Conceptualising empowerment as worldmaking, and disability as something that is ambiguous, we challenge the assumption that the aim of empowerment of disabled people is to improve their (disabled people’s) quality of life.

**Method:**

This article relies on research data (collected between January 2014 and January 2017) comprising 72 interviews and participant observations from 27 persons with disability, and 13 social workers and senior government officials.

**Results:**

We conclude that discourses of empowerment of disabled people frame disability as loss and tend to conceal the personal stories and survival operations of disabled people.

**Conclusion:**

Empowerment discourses ironically provide the platform for local power elites to ‘ride’ to fame on the backs of disabled to extend their influence in society. In the current neoliberal environment of unequal access to opportunities, disabled people must ‘play along’ as a survival strategy. Our qualitative data provide opportunities to reflect on the tensions between the ‘local and the global’, thus indicating how disability issues intersect with other wider questions.

## Introduction

Discourses of empowerment of disabled people frame disability mainly in terms of deficit, and they (discourses) tend to conceal the personal stories and survival operations of disabled people (Balcazar et al. 2012; Block et al. 2015b; Charlton 2000, 2016; Purdue & Howe 2012). Yet, when critically observed, empowerment discourse ironically provides the platform for local power elites to ‘ride to fame’ on the backs of the vulnerable – the poor, the disabled, widows − to extend their influence in society. This concurs with Shakespeare’s (1994) statement that the image of disabled people as ‘powerless’ becomes only meaningful if it occurs side by side with the benevolence of powerful elites. In the current neoliberal environment of unequal access to opportunities, disabled people must ‘play along’ as a survival strategy. But it would be misleading to (mis)interpret this strategy in terms of powerlessness. Thus, we shall adapt the notion of ‘paradox’ to open lines of discussion of how a sociocultural practice that (re)inscribes the ‘powerless’ image of disabled people while strengthening the influence of local elites could be framed as ‘empowering’ to disabled people.^1^

This article explores discourses that shape disability support among the Igbo people of Southeast Nigeria, focusing on relations between the elites of Igbo society and people with disability and on how this relationship impacts our understanding of empowerment. Overall, this article explores the inconsistencies between discourse of empowerment of disabled people and disabled people’s lived experience of disability. Through a perspective of disability that privileges resistance rather than suffering and lived experience of disability instead of pessimism (Block et al. 2015a:361), this article frames disability differently, as a ‘regenerative force’ instead of shutting down of possibilities (Block et al. 2015b). Instead of thinking about disability in terms of binary opposition, pathology or ‘loss’, our goal is to present disability in terms of full range of experiences that are shaped by sociocultural values, personal context, a family’s economic situation, environmental barriers and everyday assumptions (Block et al. 2015b:4). It should be noted that the term ‘empowerment’ is used loosely in this article, and hence this article is not about empowerment of disabled people but rather about the use of the discourse of empowerment.

The research that informs this article derives from a broader ethnographic project involving interviews, observations and archival studies; embedding in the networks of the disability community; and visiting rehabilitation centres and government departments, including attending ‘empowerment’ programmes. The fieldwork was conducted by the first author between January 2014 and January 2017 among the community of people with mobility disabilities in Southeast Nigeria. The research sought to ask a broader question about how embedded cultural notions about disability connect with the discourse of empowerment of disabled people and how this idea of empowerment plays out in Southeast Nigeria. For the purposes of this article, we are ‘reading’ empowerment discourses through the lens of Igbo worldviews.

Southeast Nigeria has a population of about 30 million people and is both culturally and linguistically homogeneous. Nevertheless, there are six states in the Southeast geopolitical zones with divergent ‘empowerment’ programmes for their respective disability community; thus, findings of this article cannot be generalised beyond the terms and local characteristics of the study participants. Second, ideas of the sociocultural forms and functions of empowerment cannot be generalised beyond the Nigerian context.

Although study participants found it difficult to communicate the meaning of *empowerment* as disability support, they often took refuge in the word (*empowerment*) when they wanted to communicate an important point about disability support, using their hands to gesticulate the sign of an airplane lifting from the ground on take-off. This is something which has been noted in the scholarly literature (Papp 2016), drawing attention to the contrast between discourses and reality.

## Disability and the struggle over definition

For Nora Groce (1999), disability is the umbrella name that unites people with diverse forms of impairment. But then disability is more than a body that does not function with 100% efficiency (Campbell 2009; Kasnitz & Shuttleworth 2001), and it is less about practical challenges than the kind of image it evokes in mainstream thinking (Devlieger & Balcazar 2010). This line of thinking provides armour to social model researchers (Barnes 1997; Oliver 1992) to argue that disability cannot exist without interpersonal relations and systems of power structure.^2^ The World Health Organisation (WHO) (2011) frames disability in vague terms as a complex, dynamic, multidimensional and (even) contested human condition. But Barnes and Mercer (2011) tend to read politics into the WHO approach, arguing that the struggle over definition is to isolate and quantify the range of challenges facing the individual for purposes of disability support. Yet the struggle over definition rages on.

However, disability has travelled a long way in public discourses since the 19th century. For example, Devlieger et al. (2003) wrote that the notion of handicap was initially invented as a name for the disability community. The term was first introduced earlier in the United States, reaching Europe after World War II. NGOs later appropriated handicap, perhaps because of the ideas of not only ‘equal chances’ but also ‘helplessness’ that it evokes. The concept of ‘disability’ was later ‘invented’ to mitigate the negative connotation of ‘handicap’. Yet those who invented ‘disability’ – less or not able – may not have foreseen the negative connotation the word would generate.

There is, thus, a sense that the notion of disability is a historically constructed concept. Pieter Verstraete (2012), following Michel Foucault (2006), believes that the framing of disability has mostly benefited from the thinking of those who are not disabled. For example, in his ‘History of Madness’, Michel Foucault identified the evolving and historically fluid nature of disability. He successfully demonstrated that what constitutes (mental) disability changes over time. Again, in the same context of intellectual disability, Devlieger (2003) documented a movement from ‘idiot’ to ‘feeble-minded’, and finally to the more acceptable ‘mental retardation’ and ‘person with mental retardation’ and currently ‘person with an intellectual disability’ in the American discourse. The system of naming and re-naming the infirmity is probably to suit the contemporary times, dissolve the difference and create a semblance of sameness between the disabled and the dominant group (Devlieger, Rusch & Pfeiffer 2003).

We shall rather argue that disability could be ambiguous, complex and an ‘unconventional’ way of being in the world that seems to offend the normative way of being in the world. Thus, disability is not a human condition that invites a simple solution.

Disability and empowerment are two sides of the same coin where one means the opposite of the other. Empowerment rhetoric was very popular among the elites and international organisations as a one-size-fits-all ‘methodology’ for fixing social and economic marginalisation of the disability community. Several decades later, disability support is still ‘packaged’ in empowerment rhetoric in the local world. It is the history of empowerment discourse that we shall now address in subsequent sections.

## Historicising empowerment discourse

Empowerment first appeared in written communication in the mid-17th-century Britain with a meaning that suggests ‘delegated authority or authorisation to act’ (Wehmeyer & Cho 2010). But most scholarly works, such as Hur (2016) and Calvés (2009), associate the most recent resurgence of empowerment discourses to the work of the Brazilian educator and philosopher Paulo Freire (1921–1997) in his ‘Pedagogy of the Oppressed’ (1993). Although many authors reference Freire’s pedagogy of the oppressed as foundational to contemporary ideas of empowerment, he did not use ‘empowerment’ in his work. Rather, the terms ‘liberation’ (used 48 times) and ‘conscientisation’ (critical reflection or awareness) were among the high-frequency words in his book. Thus, these scholars probably referred to Freire’s thoughts on ‘pedagogy of the oppressed’, firstly, as an example of how empowerment might be operationalised, and, secondly, to his audience (the poor, marginalised segments of society), as the target audiences of empowerment discourse. There are yet other sources of empowerment discourse. Calvés (2009) wrote that current ideas of empowerment also benefited from feminism, liberation theology, Black Power movement in the United States and Gandhism. Along that line, Cornwall and Brock (2005) link the history of empowerment to *Christian rights movement, New Age self-help manuals* and *business management*. Yet again, none of these historical sources specifically included the word ‘empowerment’ in their discourses that deal mostly with self-reliance. Rather, ‘empowerment’ was just an idea or interpretation of those discourses, suggesting that in these discourses empowerment was probably a label to give meaning to an idea.

In the last three decades, empowerment was the ‘in’ word among international development organisations, and other state actors, as it became the focus of many studies in the international development community. Such was the popularity of the concept that funds were committed to studies (e.g. Hennink et al. 2012; Jupp, Ibn Ali & Barahona 2010; Narayan 2002) on evaluation and measurement of empowerment as a ‘methodology’ for speaking for oneself. Although empowerment has been a topical issue since the 1990s, scholars have only recently begun to critically (re)examine its claims for the quality of life of those found at the margins of society. This effort was initiated by the work of Cornwall and Brock (2005), Cornwall (2007a), Cornwall and Edwards (2010) and Eade (2010). The following three illustrations complement these efforts.

Firstly, since 2000s most empowerment researchers have begun to think differently about empowerment. For example, Forrest (2000) suggested that ideas of empowerment had gained such purchase in the public imagination that they now appeal to the interests and purposes of both the ‘rich and powerful and poor and powerless’ alike. Pettit (2012) described how empowerment functions at the level of rhetoric especially to spice up policy documents and public communication among powerful elites while preserving the interests of the same elites along the structures of power relations (Foucault 1982).

Secondly, empowerment rhetoric is often expressed as ‘social medicine’ and a ‘methodology’ for exiting poverty and social and economic marginalisation (Eade 2010; Sugar 2015). There is a sense that ‘a dose’ of empowerment is needed to ‘cure’ or reverse social and economic exclusion of those at the fringes of society. However, Cornwall and Edwards (2010) argue that this approach amounts to oversimplification of the multidimensional issues of both poverty and economic marginalisation.

Lastly, Baistow (1994) posits that the extant literature often frames empowerment as an ‘external force’ or as something that someone does to others to ‘empower’ them. Thus, there are those who ‘empower’ others – social workers, health visitors, nurses, community psychologists, politicians, local elites and wealthy people, and those who receive empowerment – the poor, clients, patients, employees and disabled people. This perception creates a sense of binary opposition in the mainstream imagination about a world divided between the powerful and the powerless, between givers and receivers. This line of reasoning also misses the point on Foucault’s perspective on power that aligns it more closely with discourse (Foucault 1982).

## Methodology

Miles and Huberman (1994:5) admonish that qualitative enterprise is more a craft than slavish adherence to ‘methodology’. This entails that the rules of common sense and innovation are the hallmark of qualitative research. The methodology of this article consisted of fieldwork grounded in anthropology, an approach that facilitates holistic access to the field through close participant-observation, occasional interviews and archival study. Study participants consisted of 28 persons who identified as disabled persons (23 men and 5 women), and 12 non-disabled persons whose work brings them in daily contact with disabled people. In total, we conducted 38 semi-formal interviews and observations (and occasional participation) involving 10 men and 4 women with disability, and additional 14 informal interviews and observations involving 13 men and 1 woman with disability. We conducted a total of 19 semi-structured interviews with 12 non-disabled persons whose work connects with the empowerment of disabled people – disability services, rehabilitation, disability support and Igbo culture about disability. The non-disabled persons comprised social workers at government ministries, a medical doctor, one director of a prosthetics workshop and his assistant, another director of a rehabilitation centre who owns a secondary school that hosts children with a mobility disability (and their assistants), a parent of someone with a mobility disability and two old men who are knowledgeable in Igbo culture. Their various roles contributed to addressing issues raised in the research. For purposes of confidentiality, and personal terms of study participants as a vulnerable population, we decided to shield their real names and (in some cases) actual location of study.

Our interviews with social welfare officers and elites are mainly related to the policy side, government efforts and how they viewed their work. We also visited a rehabilitation centre (for persons with mobility disability) and government ministries overseeing disability issues, where we observed and asked different sets of questions relating to services for people living with disability. During interviews with senior government officials or big politicians, we often listened attentively and made a few notes because we did not receive permission to audio record. One senior civil servant in the Ministry of Education cancelled an interview when a request was made to audio record him.

Initially, new study participants were recruited through a snowball technique: one participant recommended someone she or he knew. We soon realised that this technique did not often work as planned because the new study participant might not be well disposed towards the research. As the research progressed, we began to make our own decisions about subsequent research participants focusing on those with sustained interest in the research, availability and willingness to share their experiences. We could meet someone with a mobility disability, and then open a conversation with him or her. If the person was interested in the conversation, then we could make an appointment and the conversation could continue. There were some study participants we met only once, and we did not have the chance to meet for a second time. Occasionally, some study participants, who did not understand the nature of the research, thought we were philanthropists, out to distribute money.

The first meetings were often awkward and full of suspicion on the participant’s side, and (often) an anticipation that the researcher had come to ‘help’. The researcher often introduced himself with a ready-made story about the research and its relevance to the disability community (Russell 2011:3), informing the listener that the project will produce data that would be made available to well-informed decision-makers about disability policies and programmes for the disability community. After this the researcher requested the research participant to introduce himself or herself. Then the interview began like a conservation with the purpose to relax the interviewee. During the interviews, carefully listening while taking occasional notes or recording the interviews (only with the permission of the interviewee) were regularly used practices.

Anthropological data analysis follows the interpretive tradition in terms of meaning construction that taps into peoples’ subjective experiences (Russell 2011). The preparations for data analyses commence in the field and are ongoing throughout a process of reflexivity, that is, making decisions consciously and evaluating them. Miles and Huberman further advise that researchers usually adapt their methodology to peculiarities of the research setting through the lens of the conceptual frameworks of the study which evolved gradually during the process of data collection. The onus is on the investigator to faithfully render the process followed in the data analysis. Focusing on incidents (what happened in the story not the story), we did an iterative (re)reading of research data files in line with conceptual leaning of the research and the research objectives. Having identified the research question, we returned to the files (interviews, observations, documents reviewed) to classify the key words in line with the research objectives and conceptual frameworks of the study. This approach makes it easier to match the data with different parts of the research objectives. In this article, our key words are the themes of ambiguity and complexity of disability, empowerment as worldmaking and the Igbo worldviews on support for those whose living condition is associated with the margins.

Occasionally, study participants received cash rewards. Although supplying data for the research, most of them did not fail to mention their personal financial challenges. One of the wealthy study participants raised issue with the first author because he did not give him some money. This sceptic study participant kept supposing that the ‘Whiteman’ gave the first author a lot of money for the fieldwork and he was entitled to a slice of the cash.

Gaining the confidence of study participants was a gradual process, and when this happens, they tend to enlist the researcher into their cause, detailing all instances and experiences of disability oppression in their area. In all cases, female study participants were more open than male study participants. Part of fulfilment in the field comes from hearing that some of the study participants nicknamed the first author ‘disability police’, that is someone who fights for the interest of the disability community.

### Ethical consideration

The self-funded doctoral research of Mr O.V. Nwokorie did not go through any procedure because the University of Leuven does not impose ethical clearance on research that is conducted without external funding. Rather, Mr O.V. Nwokorie took specific steps to adhere to ethical guidelines of anthropological research as outlined in American Anthropological Association ethics guidelines for field research on the use of human subjects as reported in the edited work of Robben and Slukka (2007). The guidelines provide for issues of full disclosure (in terms of competing interests), informed consent and protection of the identity of study participants. These were all observed during the conduct of the research. Mr O.V. Nwokorie travelled to the research site with a letter of introduction provided by Prof. P.J. Devlieger and copies of the consent form. The consent form proved to be an obstacle during the fieldwork because the study participants viewed it with suspicion. As a compromise, the consent form was verbally explained and agreed upon by the study participants.

## Situational context: Igbo people of Southeast Nigeria and the Igbo worldview (*uwa **Ndigbo***)

The practice of empowerment discourse links with Igbo culture. The Igbos of Southeast Nigeria are well-researched people (Cookey 2005; Iroegbu 2005; Nwagbara 2007; Stevenson 1985). Nwagbara (2007) outlines three different senses of the word *Igbo*: firstly, the Igbo territory; secondly, the domestic speakers of the language; and finally, the language spoken by them.

Although Igbo people have experienced some continuities and changes since their first contact with the larger world, there are certain aspects of their worldview that are resistant to change. As we show here, there are few aspects of everyday life that are not influenced by the local worldview. The concept of worldview or cosmology opens lines of discussion about how people make sense of the world around them including their relationship with neighbours and their natural environment. Viewed from material, spiritual and sociocultural ramifications, the Igbo worldview is given meaning by Igbo cosmology which provides an explanation for how the world is ordered. Nwagbara (2007) and Nwoye (2011:306) conceptualise Igbo cosmology or worldview as a system of prescriptive ethics, which define what the Igbo ought to do and what they ought to avoid, including the system of action which reveals the codes of conduct. Thus, worldviews are ‘products of experiences so pregnant with drama’ that such experiences give rise to symbols or totems of some sort. The worldview helps to make sense of reality, events, space-time, the environment and how to exert control over them (Nwoye 2011:306). Nwoye elaborates:

[…] a worldview can be understood in terms of a unified picture of the cosmos explained by a system of concepts, which order the natural and social rhythms, and the place of individuals and communities in them. In other words, a worldview reflects people’s basic assumptions and perceptions of the world, which give orientation and value to their lives. A people’s worldview stands for their source of explanations for the ways things are in the world, including their theories of illness, death, and misfortunes, and how human afflictions and problems can be resolved. (p. 306)

Nwagbara (2007) modified it a little through the introduction of spiritual dimension and ‘prescriptive ethics’:

The Igbo world, *uwa Ndigbo,* viewed from the material, spiritual, and socio-cultural ramifications, is made intelligible to the Igbo by their cosmology, which explains how everything came into being. It is through their cosmology that the Igbo know what functions the heavenly and earthly bodies have and how to behave with references to the gods, the spirits, and the ancestors […] two aspects of the Igbo cosmology namely: cosmology as a system of prescriptive ethics, which defines what the Igbo ought to do and what they ought to avoid; and cosmology as an action system, which reveals what the Igbo do as manifested in their overt and covert behaviour. (p. 103)

In general, a worldview is rooted in cultural assumptions or what Clifford Geertz (2009:164) considers as a web of significance that man has spun around himself. The notion of assumption implies a shared unifying conception of a given society or group through which that society or group makes sense of the world around them (Salazar 2013; Vaughter & Alsop 2017:130). The worldview represents the basis for action, social relationships, and provides explanation about everyday occurrences. It ensures the maintenance of stability in society while guaranteeing the setting of boundaries of social mores. The Igbo worldview has a bipartite structural relationship between the spirit world and the world of the living. That worldview is founded on the assumption of a dual traffic between the two worlds. Igbos consider it inevitable that those living in the world of the living are expected to transit to the world of the spirits. However, when someone joins the ancestors too early, then the people worry and attempt to make enquiries.

The Igbo worldview views the world as something that is in process of attaining equilibrium, and not a ‘natural order that goes according to a master plan’. Consequently, deities, spiritual forces as well as human beings and social relations are subject to manipulation. Nwagbara (2007:104) wrote that occurrences, such as excessive rainy season, drought, business failure, ill health or whatever threatens the life or security of an individual, are ‘interpreted by the Igbo as a sign or warning that things must be set right before they get out of hand’. As we discuss later disability is one of such occurrences with abiding significance among the Igbos.

## Disability in Igbo culture

There is no Igbo language equivalent for ‘disability’. A person with impairments (*nkwaru*) is either addressed after the impairment type (e.g. the blind person ‘*onye ishi*’, the lame-disabled ‘*onye ngworu*’, the mentally disabled ‘*onye ara*’) (Ingstad & Whyte 1995:7), or may also be addressed by the generic name of impairment – ‘*onye nkwaru*’ (a ‘dented’ or ‘unwholesome’ person)^3^. The importance of ‘wholeness’ in Igbo cosmology means that congenital disability carries a stigma.

Igbos view an impairment as a ‘misfortune’, especially where the aetiology of that impairment has spiritual roots (Ingstad 1999:757), but they do not discriminate against disability explicitly. However, they place different values on people who have and do not have a disability. In extreme cases, the Igbo worldview (*uwa ndi Igbo*) regards people with disabilities and their possessions, including the words they speak, as ‘dented’ (*asosara ya ihe*) – such as might be found on a car after an accident.^4^ Hence ‘one cannot envy a person with disability, even if wealthy’ (*anaghi agbabiri ya okpo*) (Eskay, Eskay & Uma 2012; Eskay et al. 2012; Lang & Upah 2008). Two elderly informants reported that someone with a disability may not be invited to officiate in a sacred ceremony because ‘their thinking is not clean […]’. Because their disability is viewed in terms of ‘impurity’ while ‘wholeness’ is a source of consolation among the non-disabled:

The disabled may be rich and richer than others, but there is a difference that invites ‘sorry’ to the disabled. As in the case of ritual libation, where a vote of thanks is required. A disabled person cannot officiate in such a program. If he does, the prayer will stand *suspended*. In *Omenala Ndi Igbo* (Igbo tradition), you cannot call a disabled person to pray for progress that would reach God. It is only a complete person that can call on God the maker of heaven and the earth. That is the way it is done in Igbo land in the past. (Ifeanyi Nwosu, interview, 17 February 2016)

The notion of ‘suspension’ is indicative of how Igbo culture places the disabled within the boundaries of a contextual identity (Reid-Cunningham 2009; Titchkosky 2009:250–251; Turner 2009). However, there is a government campaign to change hearts and minds on how mainstream culture relates to the disability community.

### Empowerment and Igbo language

There is no one-to-one translation of *empowerment* in Igbo language. The two closest words *nwulite* – which translates as ‘lifting-up’ (just as when the air plane lifts from the ground on take-off), and *nkwado* – a kind ‘support’ or ‘help’, play leading roles in the conceptualisation of empowerment. The disability community prefers *nwulite,* believing that education, a job, and money can supply this kind of *lifting power.* This frames empowerment as an external force or as something that is done to someone by others to ‘empower’ them (Baistow 1994).

However, the mainstream conceptualisation of empowerment as *nkwado* seems to align with the Igbo worldviews about supporting someone whose living condition is associated with the margins. For example, a senior staff of a government ministry defined ‘empowerment’ as ‘an assistance the society gives to someone with a disability because of the recipient’s limitations or inability’.

Study participants were asked questions such as ‘what comes to your mind when you hear the word “empowerment”?’ Their responses aligned with ‘what someone does to someone else’ (Baistow 1994), such as sponsoring someone in a business, offering them money or jobs and so on.

## ‘(Re)-reading’ empowerment

One might be misled by these responses to conclude that the respondents lacked the capacity to act (Balcazar et al. 2012; Charlton 2000). These responses seem to undermine other sources of data on how disabled people resist oppression and try to assert themselves in everyday life. For example, when we listened to some of their personal stories, we often ‘read’ self-assertion, civil disobedience and resistance against oppression (Balcazar et al. 2012; Charlton 2000, 2016; Freire 1993). Citing the case of John, we show below that the general views on empowerment expressed above are not always accepted in practice by those in the disability community who are on the receiving end of sympathy and ‘empowerment’.

John was born about 65 years ago, before the current advances in Western medicine in Igbo land. He was born prematurely and wrapped in bandages for 7 months in an incubator. The treatment curtailed blood circulation to parts of his leg. Thus, his right leg looked weak and the other leg seemed lifeless, and presently, he uses a walking stick to support his wobbling legs. He has a primary education and works in the local government as a messenger. Although he boasted that there was *no* question about disability that he could not answer, we could tell when he was not sure of his response to a question, for example, when he dismissed a question about the United Nations Convention on the Rights of Persons with Disabilities [UNCRPD] (United Nations 2006). Although John argued that disabled persons deserve ‘sympathy’ from the society, he was nevertheless very assertive and often spoke his mind in public. This informant gave an insight into his gradual development:

When I realised that people do not have respect for disabled people, I started dressing very neatly. Then one day:

I went to see the Chairman [sic] of our municipal council. The receptionist tried to frustrate me, saying that the Chairman was busy with someone. After waiting a long time, I decided to let myself in. I said to myself, ‘since the Chairman is in a meeting with a human being like myself, I must go and join them’. When I opened the door, I was surprised to find the Chairman alone. He too, was shocked to see me there. (John, interview, 12 January 2016)

John’s story and experience indicate the original nature of empowerment discourse operationalised as a product of ‘critical awareness’ or ‘conscientisation’ (Freire 1993:35–37), ‘resistance against oppression’ (Charlton 2000:5), ‘survival operation’ in the context of disability (Charlton 2016:59) and civil disobedience as in ‘Occupy Wall Street’ (Block et al. 2015b:9). John also observed that many young persons with a disability were struggling because they did not want to ‘learn’ from experienced disabled persons like himself. He considers himself as ‘expert’ in disability experience, especially in tackling elites:

If *big men* (rich men) want to give a disabled person something, they make it difficult. But I do not get discouraged. I follow them up persistently until they get tired. I have many strategies of following big men. For me, there is time to speak out loud and there is time to pretend as if I don’t know what I am doing. (John, interview, 12 December 2016)

In that short exchange, John summed up his vast experience of coping with disability including his tenacity in the pursuit of disability support or empowerment. Disability is, thus, a site of knowledge production and development of new skills. In the next section, we present an ethnographic account of the relation between elites and disabled people and how it mediates our local understanding of ‘empowerment’.

## Ethnographic findings

In Nigeria, empowerment is the latest container word (a lexical item with chains of equivalence or large semantic field) in their political lexicon. The word came with the latest onset of democracy in 1999. The rise of empowerment has two sources, one local and the other international. The local source was the emergence of new elite politicians competing for the heart and soul of electorates. To win elections, politicians deployed impressive and often confusing words on the campaign trail. The international connection with empowerment came with increased activities of the UN and international NGOs. Central to empowerment discourse was the widely circulated International Monetary Fund (IMF) Poverty Reduction Strategy Papers (Cornwall & Brock 2005:1045). The document was crafted in optimistic tones with lexical items that appeal to those whose living condition is associated with the margins. From then on, the word caught on and became an everyday word especially among politicians and the person in the street, further highlighting the dialectics between the ‘global and the local’ (to be addressed in the conclusion).

Nigerian political elites frequently frame disability support in terms of ‘empowerment’. ‘Empowerment’ activities diffuse over various stakeholders using various strategies and creating new spaces. Recently, the governor of a state framed his commercial vehicle loan initiative for loyal party supporters as ‘Governor […] Youth Empowerment Program’. In another case, a local politician introduced ‘medical empowerment’ for residents of his local constituency. The ‘medical empowerment’ is a 1-day free medical consultation for members of his constituency. Wives of politicians (especially governors) occasionally distribute prosthetic limbs for free to a select group of mobility disabled people. As we elaborate later, politicians organise empowerment to bring development directly to the people, to ensure that everyone benefits from the government or from the politician organising the empowerment event. Most politicians use the end of their term in office to organise empowerment for their constituents, to remind people that another election is fast approaching. In the following, we have itemised the spaces of empowerment discourses in Igbo Nigeria.

## Political campaign tool

**FIGURE 1:**
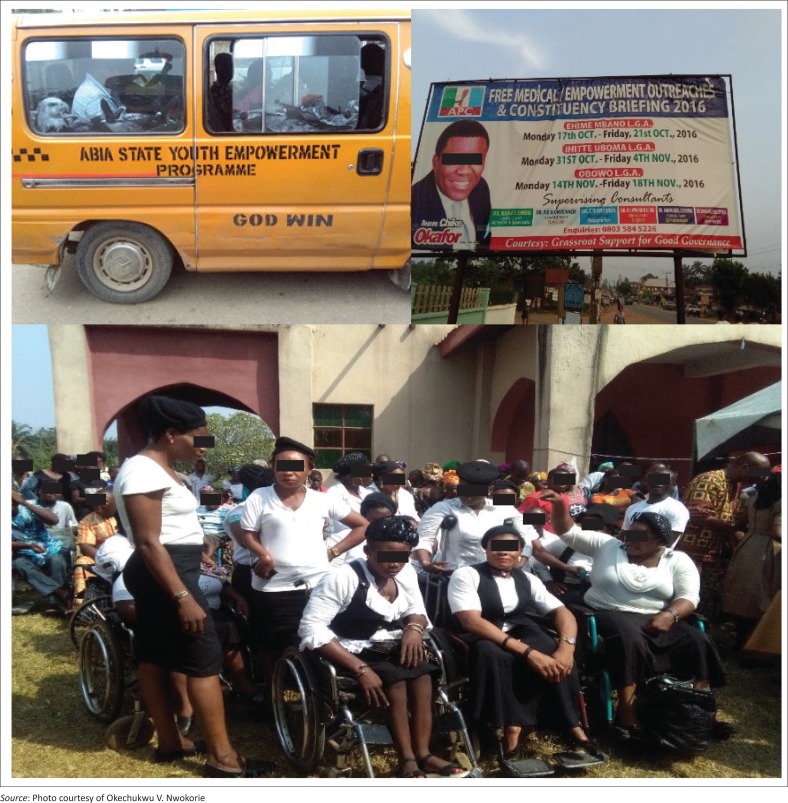
Youth, medical and private-initiative ‘empowerments’ programmes.

In Nigeria, empowerment discourse is most popular in the arena of politics. Thus, politics is currently the biggest ‘business’ in Nigeria because winning an election comes with unfettered access to surplus public finances given the thinking there that public finance does not belong to anyone. From the local municipal election to bigger state and nationwide elections, politicians do not ‘leave any stone unturned’ during elections. Extremely wealthy elites, locally referred to as ‘money bags’, often sponsor young and promising men and women into elective offices. There is often an understanding that the young politician would protect their interest on winning elections. That is why political elites also organise empowerment for the general population and disability community during the approach of general elections. Below, we give an ethnographic account of the relation between elites and disabled people and how it mediates our understanding of empowerment (Figure 1).

Ebere is a dark-skinned, middle-aged man of 40 years living with a mobility disability because of impairments in his right leg. During this meeting, he wore a handsome look in faded ill-fitting brown trousers, leaf green shirt and a light orange winter overall. On his feet were a pair of brown boots that belonged to the Nigerian Youth Corps – young Nigerian university graduates in compulsory 1-year National Service.^5^ His village was a rural deserted community located on rocky terrain. He volunteered to share some of his experiences of an ‘empowerment’ programme. When first contacted on the phone about this meeting, the energy from his excitement was obvious. His first question then was, ‘where can I meet you?’. ‘Where can I meet you?’ meant that he took the initiative, although he had problems with walking. It seemed that he was anticipating some disability support.^6^

However, the first author responded that he would rather come to meet him. Ebere was eating spaghetti mixed with a milky substance when the first author arrived in his family compound the next day. There was another man standing at the background – probably his bodyguard.^7^ Ebere’s compound was built on a stony terrain. It was made up of a group of weather-beaten houses on either side of the narrow path. Ebere’s family house was hidden on the right side of the compound. It was a mud house coated with cement and roofed with corrugated iron sheets. The meeting took place under a tree in front of his house where he erected his ‘shop’. His wares were displayed on two tables. They consisted of light articles like biscuits, sachets of Cowbell milk, groundnuts, spaghetti, cigarettes, local gins mixed with roots in two bottles, three bottles of 3-3 lager beer with two bottles of 7UP soft drink in a small bucket. On the ‘entrance’ of the ‘shop’ were worn out clothes displayed on a line of rope.

Ebere belongs to the network of members of the disability community who followed information about ‘empowerment’ programmes. His attendance at such events depended on certain calculations about means and costs of transportation to the venue of the programme. Ebere had benefited from many ‘empowerment’ programmes, but the most memorable was the one organised by the ruling political party, shortly before a recent general election. The local ward chairperson of the political party, who lived across the road from his compound, selected him for the ‘empowerment’ programme. This was how Ebere narrated his experience of the empowerment programme:

When I reached Mata Delarosa (venue of the programme), I saw many people entering there. Then I entered the big church and prayed. I said, ‘Oh God you know the reason they have invited me here. Help me today to get something good today’. When I came out, I saw many things: grinding machine, tricycles, generators, motorcycles, tailoring machines, wheel barrows, and other items. Then after some time a member of the Federal House of Representatives (elected politician) came and encouraged us to continue to support the ruling party. Then they started calling names of people beginning from university graduates. After that, came the turn of people doing private business who did not have enough money to run their business. Each name was called and given a bank cheque. The first group was given 100,000.00 naira (about 250 EUR). Then second group received 50,000.00 naira (125 EUR). Then they called my name and gave me a cheque of 50,000.00 naira. I was so excited that I almost lost the cheque. Then my brother followed me to cash the money from the bank to buy some things needed to complete my marriage to my wife. (Ebere, pers. comm., 26 February 2016)

The empowerment programme was a win–win situation for the political party and the recipients, especially given the approaching elections. Although the political party was hopeful that recipients of the empowerment would reciprocate them during elections, the money Ebere received afforded him the resources to complete his marriage rites. As someone whose ‘one leg does not touch the ground’ (as he reported about himself), it was important that he married a lady who would complement his disability.

In contrast, 35-year-old Edwin, who comes from the same village as Ebere, wondered why his name was omitted in the empowerment programme for which Ebere received money. By that he seemed to be questioning the criteria used to make the selection for beneficiaries. However, the question goes deeper than that because it was representative of all empowerment programmes studied during the fieldwork. However, in the case of this empowerment programme, it was at the discretion of the person doing the selection to nominate their preferred candidates.

It seems that Edwin had been losing out in empowerment programmes, or he had been attending the wrong ones, because he recounted an experience of ‘empowerment’ in which he was only served snacks and Coca-Cola and told to go home. Although a lot of items were displayed, the distribution was marred by confusion:

On that day, no one took us seriously. If you do not have someone in government, no one listens to you. Many disabled people came, and many things were made available, but only those who have a representative in government received something. At the state capital, the organizers told us that our own share of empowerment would be sent through our local traditional ruler. After that, we didn’t hear anything about the money. We only got Coca-Cola and about 15 of us received one bag of rice with a few tins of tomato. (Edwin, pers. comm., 28 February 2016)

For disabled persons such as Edwin, many factors still count against them. For example, he did not belong to the organised disability movement. He was one of the people who prefer to isolate themselves from the organised disability community because of the organisation’s financial demands, especially the monthly dues. They are cut off from the flow of information circulating within the disability community. The network of the local Disabled People’s Organisations (DPOs) enhances circulation of information through the mobile phone. Information technology makes the disability movement very mobile in mobilising themselves for ‘empowerment’ programmes.

The Speaker’s empowerment is another example of the use of empowerment as campaign tool. The organiser of this empowerment was the Speaker of the State House of Assembly. This empowerment was a response to the earlier one because it was organised by a rival political party. He staged the empowerment towards the end of his tenure to hint to his constituency that he was still in politics. To ensure an equitable representation, recipients of his empowerment programmes were composed of selected individuals from each of the communities within his constituency, market women, town unions and organised disability groups. Incidentally, this empowerment raised so much controversy and remained unresolved while the first author was in the field. There were lots of complaints by disabled and non-disabled people who reported that they were omitted in the sharing of the money. However, the focused local disability organisation refused to be intimidated. This is how the secretary of the local disability organisation described the event:

We received a letter that the Speaker (of the State House of Assembly) was going to empower people. The letter said that we need to make our own application so that we can benefit in the program. Then we made our application. We came to the venue of the program (local government headquarters) before 10h00. Many things had been made available before then. About 13h00 the MC (Master of ceremony) announced that the speaker would soon arrive with commissioners. When the speaker arrived, his commissioner friends praised him (the Speaker) that he is a true son of this community. That was why he has come to empower the poor, widows, the youths and disabled. Then people asked the speaker about his next political ambition, but he refused to answer that question. The MC began to call a list of names of people to be empowered from community to community. Then he called us (the disability community). I came forward and received an envelope […]. (Magnus Opkara, interview, 10 December 2016)

Then he took the envelope with a cheque for 500 000 Naira (about 1200 EUR) home and later secured the money in the bank. Soon the money became the source of acrimony within the local community. This was because everyone became interested in the money, especially those who did not belong to the organised disability group. The social welfare officers who worked at the local municipality wanted to supervise the sharing of the money but the local DPO refused. Many people felt offended because they were omitted in the sharing of the money. But perhaps the money generated some dust because disabled people were involved in the sharing of the money. There was probably a sense that they were incapable of organising things properly or because they organised the sharing of the money equitably. The first author stumbled on this story late during the fieldwork on the day he visited the social welfare officer at the local municipal council. The social welfare officer was not in the office. Then the receptionist directed him to another social welfare officer who uses a wheelchair, and whose perspective added value to the controversy surrounding the Speaker’s empowerment money. He (the wheelchair user) was the secretary of the local DPOs during John’s tenure, and must have lost out in a power struggle. But the first author did not form those thoughts into words. When the first author probed deeper, a few of his grudges were divulged:

There is problem with the authorities and there are issues with the disability community as well. Earlier, there used to be some understanding between the DPOs and the social welfare office here, but the way the Speaker’s empowerment money was shared created lots of problems. The government does not seem to pay any attention to their plight, and again, the disability community is often violent because they often feel cheated. (Clement Ogu, interview, 12 December 2016)

Orji, the Secretary of the local DPO later, narrated the genesis of the quarrel in more detail. He accused the social welfare officer of short-changing them in the sharing of bags of rice they received at one empowerment event. Secondly, the DPOs argued that the welfare office did not play any role in the application that yielded the Speaker’s empowerment money. Apparently, the social welfare office saw themselves as stakeholders in the sharing of the Speaker’s empowerment money. They had hoped to get a share from the money whereas the money was a donation to disabled people.

Currently, the disability community members were paying a price for their new-found ‘independence’, as they currently organise their things without input of the welfare office. Previously, the local social welfare office sponsored their ‘empowerment’ trips and benefited from proceeds of those trips. But all that changed after the Speaker’s empowerment money was received and shared without the input of the social welfare office.

From local experience, the social welfare office had sought to go beyond supervision of the activities of the local DPO to ‘colonise’ them by, for example, deciding on the mode of sharing the speaker’s donation, and telling the disability community how to run their organisation. The thinking that disabled people ‘need help, and protection’ (Shakespeare 1994) means that the social welfare officer forgot that many members of the local DPO were heads of families and fathers who make decisions in their homes. One study participant reported that disabled persons know who respects them and who does not. What counts as ‘empowerment’ or disability support is not the primary source of income for most disabled people, but an additional source to agument their regular sources of income. But everyone (disabled and non-disabled) is interested in that additional income.

## Extension of the Igbo worldview on support for the less privileged – *Oji ngaji eri cheta onye ji aka*

*Oji ngaji eri cheta onye ji aka*, a proverb that means ‘those eating with an iron spoon should remember those eating with hands’, is Igbo culture-sensitive means of helping those living at the margins of society.

Empowerment, at the level of the state, is an occasion to first label the disability community as the ‘less privileged’, and then show that the government cares for people with a disability. For example, a civil servant in one government ministry overseeing disability issues reported as follows:

When the government decides to do ‘empowerment’ for disabled people, the commissioner of the relevant government ministry receives a circular from the office of the governor. Then the government publicises the event through the electronic media. They also send invitations to organised disability groups through the welfare offices in the local Municipalities. The local Municipalities also organise the transport (often buses) for the disability community attending the event. (Ignatius Onyegbula, interview, 18 November 2016)

After the governor authorises the ministry responsible for disability issues to ‘do empowerment’ for the disability community, the Accountant-General releases money to the ministry officials who place orders for the material items, such as cooking stoves, grinding machines, wheelchairs and walking sticks. However, one informant complained that the disability community is rarely consulted at the planning stage of ‘empowerment’. Again, most of the items purchased were of sub-standard quality.

Before the start of the empowerment programme, government officials make speeches, in which they first ‘console’ the disability community (also known as ‘special citizens’) and tell them to be courageous in their ‘adversity’. Secondly, they remind them that the government has not ‘forgotten’ them. After the speeches, a member of the disability community often gives a vote of thanks, praising the government for remembering ‘disabled people’. After that, food and drinks were served.

Usually, those who attend government empowerment programmes outstrip available provisions. Thus, the commissioner in charge of the government ministry uses her or his discretion to draw a list of beneficiaries in advance. In principle, the ministry holds a file with names of applicants who had earlier requested disability assistance; however, the commissioner, politicians, senior government officials, persons connected to the governor, elite disabled persons and executives of the organised disability community also have their own ‘candidates’ who need these empowerment materials. At the end of the programme, the same persons who already own wheelchairs also receive more wheelchairs to resell them to those who were not ‘visible’ enough to receive any.

### A chat with a stakeholder

During fieldwork, a senior government official in the ministry in charge of disability matters mentioned that their stores were filled with wheelchairs donated by the Japanese government and 40 wheelchairs and 10 tricycles received from Agip petroleum. He was awaiting directives from his bosses before distributing the items. In response to a question about how he intended to distribute the donations, he replied that those who donated the items gave specific instructions about how to share them – to airports, hospitals and persons with disability. But he confessed that his superiors had ‘tied his hands’:

I went into my file and saw some applications for wheelchairs. I gave it to my staff and told them to compile these names. Then I started reaching out to some people who I knew. I have about 20 names of genuine people I know who need these things. My intention was to write their names down and call them and give them the items. But administratively, I am under somebody. When I presented this file to my bosses they told me to wait. When I get these kinds of things, I pass the information to my bosses and they must direct. You can only act when you get their response. That is why some of these things are there, because I cannot take actions on my own and invite them to come and collect. (Ogechi Onuoha, interview, 22 March 2016)

Among the persons contacted for the wheelchairs was a certain woman with a mobility disability whom they encountered in a village when he and his boss (the permanent secretary) went to pay salaries of civil servants. On that occasion, the lame-disabled woman could not access the two-storey building to receive her salary. The people helped carry her to the top floor. ‘She did not own a wheelchair’, said our informant, ‘my boss directed me to take her name and phone number so that any time we are sharing wheelchairs, we shall give her one’.

But when he spoke to his boss (the permanent secretary) about the donated items, she replied that the commissioner and the governor’s wife were interested in them. What the permanent secretary meant was that the wheelchairs and tricycles would not be distributed without adequate and elaborate publicity. The aim of the publicity was to show that the state is a caring father of the disability community, although the donation came from foreign governments and multinational corporations. The challenge with this elaborate publicity means that elite disabled people, people with ties to government, loyal party members would receive priority in the distribution of the mobility aids at the expense of rural little known, uninfluential disabled people. Then our informant promised to inform us about the final decision on how the wheelchair was finally distributed:

Many people have been calling me to know when they will receive their mobility aids. What I am trying to think is no matter when the distribution will commence, I will still refer to this list. I will tell them that there are some people who genuinely need these things. When you publicise it, you see that there are people who already have and who would still come to collect to add to the ones they have. Especially, these big disabled people who are known. When they collect, they go and sell. (Ogechi Onuoha, interview, 26 March 2016)

In their scoping study of disability issues in Nigeria, Lang and Upah (2008) have nicknamed the ‘big disabled people’, as ‘disability elites’. They are (often well educated) people with a minor disability who are well established within the community, and whose disability constitutes little or no hindrance. Yet, this category of persons often exploits their disability to maximum advantage, often acting as representatives of those categories of people with serious disability and who live in the villages. Thus, reference to ‘big disabled people’ also helps us to understand the phenomenon of disability intersectionality (Goodley 2013).

## Conclusion

Discourse is a means of dealing with a phenomenon through words, written texts, institutions and everyday practices to realise certain assumptions about the phenomenon and to construct it in a particular way (Ingstad & Whyte 1995:19). In Nigeria, discourses of *empowerment* of disabled people coincide with specific cultural assumptions about disability, prevailing culture-sensitive responses to those at the margins of society, and quest for power and prestige among local elites. Thus, disability is something that is both productive and socioculturally functional. The concept sets in motion a process of redistribution and exchange but sometimes also of disappointment. The concept of disability becomes instrumental for the political elite and for the disabled informed elite alike. It becomes an arena of ‘power play’ on which powerful elites superimpose their authority over those the society has labelled as ‘different’. This instrumental role implies that disability is a cultural space that does not only take from society but also gives back something in return. Despite these complexities of disability, the image of disability is still trapped in the ‘prison’ of prevailing ideas of otherness and liminal figures.

Empowerment research has made meaningful contributions to scholarship. This article has abstained from engaging in a debate about what empowerment is supposed to mean or to privilege one definition of the concept over another, or even to downplay the efforts of dedicated scholarship in empowerment research. Such pursuit would have been fruitless and unproductive. To do so would have been following a limited approach to the objective of this article – relation of local elites to disabled people.

However, empowerment discourse in general is increasingly losing the ‘goodwill’ of many scholars (Cornwall 2007b; Cornwall & Brock 2005; Cornwall & Edwards 2010), and slipping down the league of ‘renegade’, ‘slippery’ concepts that serve to ‘energise’ positive feelings about the idea of an imagined world (Sugar 2015). For example, in April 2017, the German Chancellor Angela Merkel organised a widely publicised event attended by women of influence from all over the world. The theme of the event was ‘The Women’s 20: Shaping the Women’s Economic Empowerment Agenda’. During this work, we did an online search on ‘empowerment’-related stories on the CNN platform, and the search yielded 649 hits on the CNN platform alone. On Google search, ‘empowerment’ returned 100 000 000 (hundred million) hits. It seems that the popularity of empowerment draws from the role of the thought or imagination in framing certain concepts which seem to ‘clothe’ the imagination with the raw material needed to create a mind picture about an ideal world in the making (worldmaking) (Ogden & Richards 1989; West 2005). For critics of empowerment discourse, the concept probably gains part of its purchase as solidarity vocabulary that tends to evoke a specific utopia that whets the appetite of those living in the margins while concealing the politics.

Discourses of empowerment of disabled people highlight some of the problematics of local−global relations where the local world appropriates global discourses into the local culture (Whyte & Ingstad 2007). Nigeria is part of ‘modernity’, but it is suspended on the balance between the global and the local, between demands of United Nations Convention on the Rights of People with Disability (CRPD) and culture-sensitive approaches to disability support. Although CRPD has a binding obligation to state parties such as Nigeria, Nigeria has no legal provisions for disability support. Policymakers often shelter behind the local approaches to disability support when it suits their purpose. Ad hoc management of disability issues seems to justify Edwin’s question about decision-making processes for disability support. The local situation is compounded by the realisation that the concept of ‘disability’ was unknown in the pre-capitalist Igbo society (Ityavyar 1987), and it (disability) has no direct translation in Igbo language because the concept (disability) does not originate in the local culture. Colonial contact introduced hybrid systems of meaning-making that left the local context in a liminal state. Reflecting on this point, Sachs (2010) wrote that:

the campaign to turn traditional man into modern man has failed. The old ways have been smashed, the new ways are not viable. People are caught in the deadlock of development. (p. xviii)

This article has shown that in the Nigerian context, empowerment aligns with aspects of the local culture about ‘helping’ poor people. In the context of disability, this idea of empowerment conceals personal stories and survival operations of disabled people such as John (Balcazar et al. 2012; Block et al. 2015b; Charlton 2000, 2016; Purdue & Howe 2012). When observed critically, empowerment discourse ironically provides the platform for local powerful elites to ‘ride’ to fame on the backs of the vulnerable – the poor, the disabled, widows − to extend their influence in the society. In the current neoliberal environment of unequal access to opportunities, disabled people must ‘play along’ as a survival strategy (Charlton 2016). However, it would be misleading to (mis)interpret this strategy in terms of powerlessness. This aspect then clearly adds a performative aspect to the disability paradox, namely that people indeed must play along, but moreover that such performance and by extension the empowerment-as-performance confirms that disability is to be understood through an album of images that includes helplessness and thus the need of non-disabled people to help out.

## Footnotes

1 *Paradox* or contradiction is a concept that highlights a situation where a proposition, or an idea or programme of action, begins with a sound, logical reasoning, but leads to a conclusion that seems contradictory. The concept has featured in researches in, for example, education (Depaepe 2008; Lam & Kwong 2012), empowerment (Botchway & York 2001) and disability studies (Albrecht & Devlieger 1999). In the field of disability studies, Albrecht and Devlieger popularised the concept of paradox in their seminal work on ‘The Disability Paradox: High Quality of Life Against All Odds’ (Albrecht & Devlieger 1999), in which the authors explored the coping mechanisms of persons with serious disabilities who reported good quality of life despite (supposedly) adverse health condition.

2 But there is also the trap of ‘simplifying’ disability solely in terms of binary opposition, ‘us’ and ‘them’ which we tend to avoid in this paper.

3 A devaluing title often used out of earshot. The plural form is *ndi nkwaru.*

4 During a radio discussion programme, which was organised during this fieldwork, no pejorative language for disability was mentioned. This was hardly surprising because the aim of the public communication is for the purposes of changing hearts and minds about disability.

5 The Nigeria Youth Corps are young Nigeria university graduates below the age of 30 years doing compulsory 1-year National Service after graduating from the university.

6 It took the study participants a long time to understand that the objective of the study was not to donate money. For this man, the opening sentence – ‘where shall I meet you?’ – seems to prove this point.

7 During the first meetings, many male study participants kept stand-by bodyguards to defend them just in case a fight breaks out. Those who could not afford hired bodyguards often relied on family members. The man remained halfway through the interview before he left.
